# Predisposing Factors for Pseudoplacentational Endometrial Hyperplasia or Cystic Endometrial Hyperplasia in Dogs and Their Association with Pyometra

**DOI:** 10.3390/vetsci12010001

**Published:** 2024-12-26

**Authors:** Clarissa Helena Santana, Monique Ferreira Souza, Laice Alves da Silva, Lucas dos Reis de Souza, André Marcos Santana, Ayisa Rodrigues Oliveira, Tatiane Alves da Paixão, Renato Lima Santos

**Affiliations:** 1 Departamento de Clínica e Cirurgia Veterinária, Escola de Veterinária, Universidade Federal de Minas Gerais, Belo Horizonte 31270-901, MG, Brazil; 2 Departamento de Medicina Veterinária, Campus Regional de Umuarama, Universidade Estadual de Maringá, Umuarama 87506-370, PR, Brazil; andrevetms@gmail.com; 3 Departamento de Patologia Geral, Instituto de Ciências Biológicas, Universidade Federal de Minas Gerais, Belo Horizonte 31270-901, MG, Brazil

**Keywords:** reproductive pathology, uterine lesions, inflammation, endometritis

## Abstract

Pseudoplacentational endometrial hyperplasia (PEH) and cystic endometrial hyperplasia (CEH) are both hyperplastic conditions that may occur in the canine uterus during diestrus. CEH can impair fertility, and, although the consequences of PEH are poorly known, this condition is significantly associated with pyometra. The aim of this study was to investigate frequencies of both PEH and CEH in female dogs according to age, size, breed, and breed group. Uteri from 300 female dogs were sampled and processed for histopathologic analyses. Lesions were identified, and frequencies were statistically compared. PEH was significantly more frequent in dogs between 4 and 12 years of age and significantly less frequent in Yorkshires than Shih-tzus, as well as in the breed group that includes Brazilian Terriers and Yorkshires. CEH was significantly more frequent in dogs older than 12 years, but no breed predisposition was observed. The frequency of PEH was significantly higher than that of CEH. The frequency of pyometra in PEH cases was significantly higher than endometritis or uterus without inflammation. This study identified age, breed group, and size as predisposing factors for PEH and further demonstrated the association between this condition and pyometra in dogs.

## Simple Summary

Reproductive diseases are quite important among dogs, especially the condition named pyometra, which is characterized by uterine bacterial infection with an intense inflammatory reaction that is characterized by the accumulation of a large amount of pus in the uterus. This may lead to a life-threatening disease. Importantly, the mechanisms that trigger pyometra are not completely known, particularly its association with other uterine changes, namely, pseudoplacentational endometrial hyperplasia (PEH) and cystic endometrial hyperplasia (CEH). In this study, we investigated the occurrence of both PEH and CEH in female dogs according to age, size, breed, and breed group and their association with pyometra.

## 1. Introduction

The interest of the scientific community in reproductive biotechnologies and the management of female dogs has increased over the past few years, with a particular focus on reproductive diseases. Uterine inflammation, neoplasms, and hyperplastic lesions, such as cystic endometrial hyperplasia (CEH) and pseudoplacentational endometrial hyperplasia (PEH), are highly relevant in female dogs [1]. Although both CEH and PEH are lesions that occur during the diestrus, they are completely different conditions [2,3].

PEH, formerly known as deciduoma [4], has been renamed and better characterized as PEH [2]. Although there is evidence that PEH is common in dogs [5,6], it is still rarely described, probably due to a lack of knowledge about the condition [5]. Grossly, PEH can be localized, forming a solitary nodular lesion, or diffuse, with irregular endometrium thickening and formation of variable-sized cystic structures [3,7]. Grossly, cases of diffuse PEH can be impossible to distinguish from CEH [3]. Histologically, PEH is characterized by endometrial thickening, with hyperplasia and hypertrophy of the luminal and superficial glands epithelia. In some cases, there may be papillary projections to the lumen. Epithelial cells of the luminal or superficial glands become columnar and finely vacuolized, which characterizes the decidual reaction, an important feature in PEH diagnosis. Superficial and deep glands can be variably dilated, and the epithelium from deep glands remains cuboidal and non-vacuolized [2,3]. These morphologic features resemble both the labyrinth and glandular placental zones, and the epithelium with decidual reaction resembles placental decidual cells [8].

CEH is grossly characterized by enlargement of the uterine horns, with endometrial thickening, multiple cystic structures, and variable amounts of intraluminal mucous accumulation [1]. Histologically, the endometrium is thickened, with moderate to severe dilatation of most endometrial glands, including superficial and deep glands alike. Depending on the intensity of glandular ectasia and the chronicity of the lesion, fibroplasia or fibrosis may be observed [1,2]. The pathogenesis of CEH is related to hormonal changes, i.e., a high serum concentration of estrogen followed by a high serum concentration of progesterone [9]. Experimental studies indicate that the intensity of progesterone serum concentration is not related to the intensity of the lesion, suggesting that the most important factor is previous exposure to high concentrations of estrogen [10]. Supposedly, estrogen induces the expression of progesterone receptors, thus enhancing endometrial sensitivity to progesterone [7].

Both PEH and CEH are conditions that can be associated with pyometra. Years ago, it was established that CEH was associated with pyometra under experimental conditions with a hypothetical cause-and-effect relationship [11]. However, recent studies demonstrated that CEH is not associated with naturally occurring pyometra, corroborating the hypothesis that CEH and pyometra may simply be co-existing conditions during the diestrus [5,10]. Interestingly, although the consequences of PEH in canine female health or fertility are not well known, it has been demonstrated that the frequency of PEH in female dogs is high and that there is an association between PEH and pyometra [5].

Considering that both CEH and PEH are potential causes of reproductive and health problems in dogs, a better understanding of the occurrence of these conditions is highly relevant. Additionally, there is no information about the distribution of PEH in dog populations or analyses of predisposing factors. Therefore, the goal of this study was to describe the epidemiological distribution of PEH and CEH in dogs, characterizing frequencies according to age, size, breed, and breed groups.

## 2. Materials and Methods

### 2.1. Animal Sampling

Samples of both uterine horns from 300 dogs were obtained from archived paraffinized tissues at the Universidade Federal de Minas Gerais (UFMG) over a period of six years (from 2017 to 2023). All samples were from dogs brought to the UFMG Veterinary Hospital for elective or pathological ovary-salpingo-hysterectomy (OSH). Data collection, including age, breed, breed group, and weight, were recorded. Breed groups were classified according to the Federation Cynologique Internationale (FCI) (https://www.fci.be/en/ accessed on 22 December 2024), which includes ten groups of breeds (Table 1). Pitbulls and mixed-breed dogs are not included in FCI group classifications; therefore, in this study, they were classified separately. The size was classified according to breed and, for Poodle, Siberian Husky, and mixed-breed dogs, according to weight. Size classification according to weight was developed according to FCI recommendation considering dogs less than 10 kg as small size, with from 11 to 25 kg as medium size and over 25 kg as large-size dogs (https://www.fci.be/en/ accessed on 22 December 2024). Pregnant dogs spayed right after the labor, with a history of recent labor or suspect pregnancy, were not included in this study.

The procedures in this study strictly adhered to all applicable Brazilian laws and regulations, and, since it was based on a retrospective evaluation of archived paraffin-embedded tissues acquired for diagnostic purposes or routine spaying, it did not require previous approval by the institutional Ethics Committee on the Use of Animals.

### 2.2. Histopathology

Samples of the uterus were fixed in 10% buffered formalin for from 24 to 48 h and routinely processed for paraffin embedding. Uterine samples were obtained from the cranial, medium, and caudal thirds of both horns. Paraffinized tissues were sectioned in a microtome (3 µm-thick sections), mounted on glass slides, and stained with hematoxylin and eosin.

**Figure 1:**
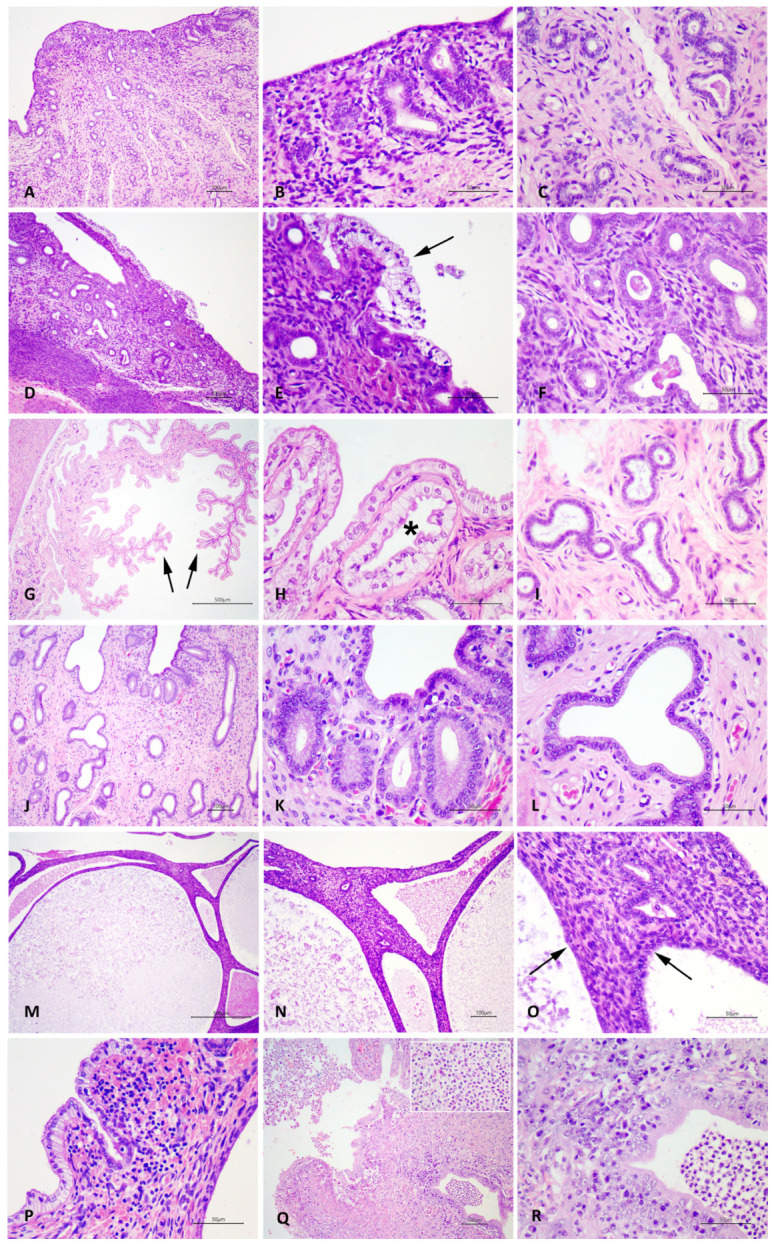
Canine uteri. Scoring criteria for hyperplastic endometrial lesions. (**A**) Uterus with a normal superficial endometrial epithelium, (**B**) superficial endometrial glands, and (**C**) deep endometrial glands. HE, scale bars = 100 µm (**A**), 50 µm (**B**,**C**). (**D**) Uterus classified as PEH score 1 (PEH S1) with multifocal areas of hyperplasia and decidual reaction at superficial endometrium; (**E**) columnar, hypertrophic, and vacuolated cytoplasm characterizing decidual reaction (arrow); (**F**) superficial and deep endometrial glands with mild dilatation and cuboidal epithelium. HE, scale bars = 100 µm (**D**), 50 µm (**E**,**F**). (**G**) Uterus classified as PEH score 2 (PEH S2) with severe endometrial hyperplasia, projecting to the uterine lumen (arrows); (**H**) superficial endometrial epithelium and superficial glands with hypertrophic epithelium, decidual reaction, and moderate dilatation of glands (asterisk); (**I**) deep endometrial glands with moderate dilatation and normal cuboidal epithelium. HE, scale bars = 500 µm (**G**), 50 µm (**H**,**I**). (**J**) Uterus classified as CEH score 1 (CEH S1) with from mild to moderate dilatation of all endometrial glands; (**K**) superficial endometrial epithelium and epithelium of superficial endometrial glands are cuboidal and non-hypertrophic; (**L**) deep endometrial glands moderately dilated and with cuboidal and non-hypertrophic epithelium. HE, scale bars = 100 µm (**J**), 50 µm (**K**,**L**). (**M**) Uterus classified as CEH score 2 (CEH S2) with intense dilatation of all endometrial glands with intraluminal mucous deposition in glands; (**N**) superficial endometrial epithelium and epithelium of superficial endometrial glands are cuboidal and non-hypertrophic with intraluminal mucous deposition; (**O**) deep endometrial glands moderately dilated and with cuboidal and non-hypertrophic epithelium (arrows). HE, scale bars = 500 µm (**M**), 100 µm (**N**), 50 µm (**O**). (**P**) Uterus with endometrial lymphoplasmacytic infiltrate, characterizing a case classified as moderate endometritis; (**Q**) uterus with endometrial diffuse and severe inflammation, with abundant neutrophilic exocytosis and accumulation in uterine and glandular lumen, characterizing a case classified as pyometra; (**R**) endometrial gland with intraluminal neutrophilic accumulation surrounded by endometrial histioplasmacytic infiltrate. HE, scale bars = 100 µm (**Q**), 50 µm (**P**,**R**).

Histopathological analyses were performed in from three to six sections of each uterine horn, following criteria described by Santana et al. (2020) [5], to classify cystic endometrial hyperplasia (CEH), pseudoplacentational endometrial hyperplasia (PEH), and uterine inflammation. Differentiation between CEH and PEH was based on morphological parameters as previously described [2]. Histological scores are described in Table 2 and illustrated in Figure 1.

### 2.3. Statistical Analyses

Frequencies of lesions and score analyses were statistically analyzed by non-parametric Fisher’s exact test and Kruskal–Wallis and Dunn’s tests, respectively, at GraphPad Prism 8.0.1 program with *p* < 0.05.

## 3. Results

### 3.1. Characterization of Dogs Included in This Study

Characterization of the population included in this study is detailed in Supplementary Figure S1. Briefly, the age of female dogs ranged from 1 to 19 years, with a median and mean of 9.08 and 9.04 years, respectively. It was not possible to classify two dogs as a record of age was not available. Size groups included small (50%), medium (22.4%), and large (27.6%) dogs. It was not possible to classify ten mixed-breed dogs as no record of weight was available. Of all 300 female dogs, 72% (216/300) were included in 38 breeds and 28% (84/300) were mixed breeds. Breeds include all breed groups except for groups 7 and 10 (Table 1).

### 3.2. Frequency of PEH and CEH

**Figure 2:**
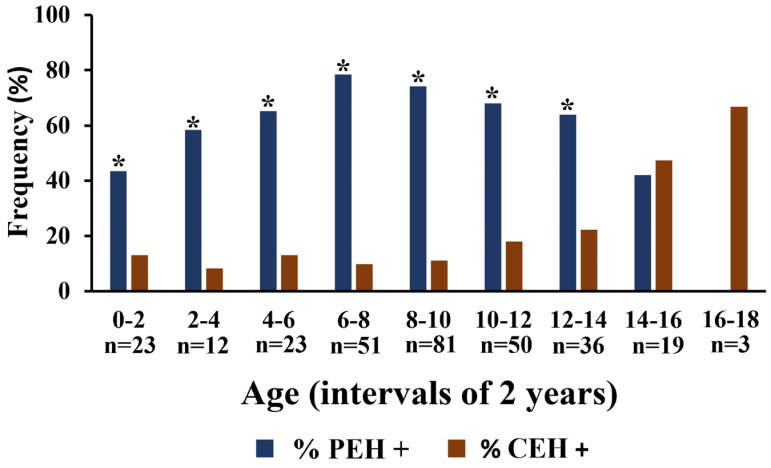
Frequencies of pseudoplacentational endometrial hyperplasia (PEH+) and cystic endometrial hyperplasia (CEH+) in dogs according to age intervals of 2 years. Median frequencies were compared in each age interval by non-parametrical Fisher’s exact test (* *p* < 0.05).

Considering all uteri included in this study, 15.3% (46/300) and 51.0% (153/300) had PEH S1 and PEH S2, respectively, totaling 66.3% (199/300) of dogs with PEH (PEH+), whereas 16.3% (49/300) of dogs had CEH, being 9.3% (28/300) CEH S1 and 7.0% (21/300) CEH S2. The frequency of PEH was significantly higher than that of CEH (*p* < 0.0001). Additionally, evaluating occurrences by age intervals of two years, frequencies of PEH were also higher at each interval from 0 to 2 until from 12 to 14 years old, with no differences between 14 and 18 years old (Figure 2).

#### 3.2.1. Frequencies of PEH According to Age, Size, Breed, and Breed Group

**Figure 3:**
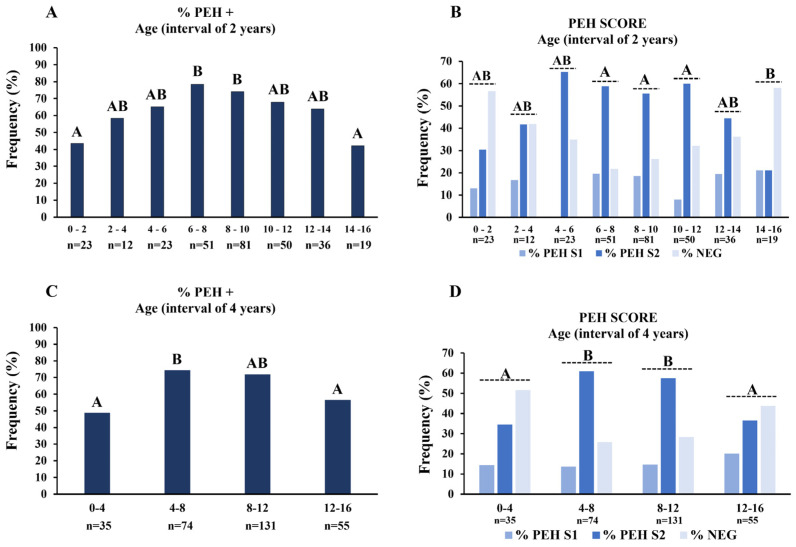
Frequencies of pseudoplacentational endometrial hyperplasia (PEH) in uteri of dogs and score analyses, with PEH score 1 (S1), PEH score 2 (S2), and negative PEH cases (NEG), according to age intervals. (**A**) Frequencies of PEH+ cases according to age intervals of two years. (**B**) Score analyses according to age intervals of two years. (**C**) Frequencies of PEH+ cases according to age intervals of four years. (**D**) Score analyses according to age intervals of four years. Medians of % PEH+ were compared by non-parametrical Fisher’s exact test, and the distribution of scores at each age interval was compared by Kruskal–Wallis and Dunn tests. Uppercase letters indicate statistical differences between age intervals (*p* < 0.05).

Intensities of PEH were classified as PEH S1, which was considered an initial and mild manifestation of PEH, whereas PEH S2 was considered an advanced and severe manifestation. However, the frequencies of PEH S1 and PEH S2 were not higher in younger dogs when compared to older dogs, which does not support the notion that the lesion progresses over the lifespan of the dog. Analyzing age intervals of 2 years, frequencies of PEH were higher between from 6 to 8 and from 8 to 10 years when compared to dogs older than 14 years or younger than 2 years (Figure 3A), indicating that PEH is more frequent in middle-aged dogs. Analyzing the score classification, higher scores were observed between 6 and 12 years of age when compared to between 14 and 16 years of age (Figure 3B). To further evaluate the frequency of PEH according to the age of the dog, animals were regrouped by intervals of 4 years. According to this age distribution, the frequency of PEH (regardless of the score) was higher from 4 to 8 years of age when compared to dogs older than 12 years or younger than 4 years (Figure 3C). Score analyses demonstrated higher PEH S2 between 4 and 12 years of age compared to other age intervals (Figure 3D). Therefore, regardless of the analytical strategy of grouping in intervals of 2 or 4 years, PEH is more frequent and more intense in middle-aged female dogs.

**Figure 4:**
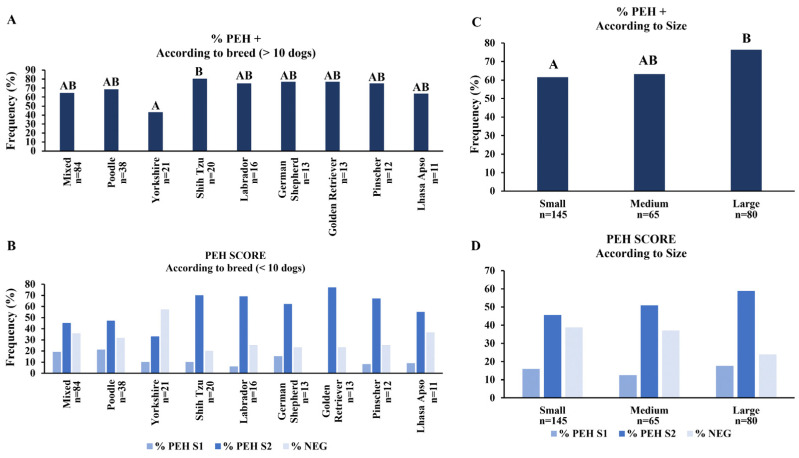
Frequencies and scores of pseudoplacentational endometrial hyperplasia (PEH) in dogs according to breed and size. (**A**) Frequencies of PEH+ cases according to breeds. (**B**) Score analyses of PEH according to breeds. (**C**) Frequencies of PEH+ cases according to size, including small, medium, and large dogs. (**D**) Score analyses of PEH according to size. Medians of frequencies of % PEH+ were compared by non-parametrical Fisher’s exact test and score distribution by Kruskal–Wallis and Dunn tests. Uppercase letters indicate statistical differences between age intervals (*p* < 0.05).

A total of 37 breeds recognized by the FCI, as well as Pitbull and mixed-breed dogs, were included in this study. Frequencies of PEH according to the breed were analyzed considering breeds with at least 10 individuals in this study (Figure 4A,B). The frequency of PEH was lower in Yorkshires than in Shih-Tzus (Figure 4A). No significant differences in PEH scores were observed according to the breed (Figure 4B). Considering animal sizes (small, medium, and large), frequencies of PEH were higher in large dogs compared to small dogs. No significant differences were observed between small and medium or large and medium dogs (Figure 4C). No significant differences were observed considering the PEH score distribution according to the size (Figure 4D).

**Figure 5:**
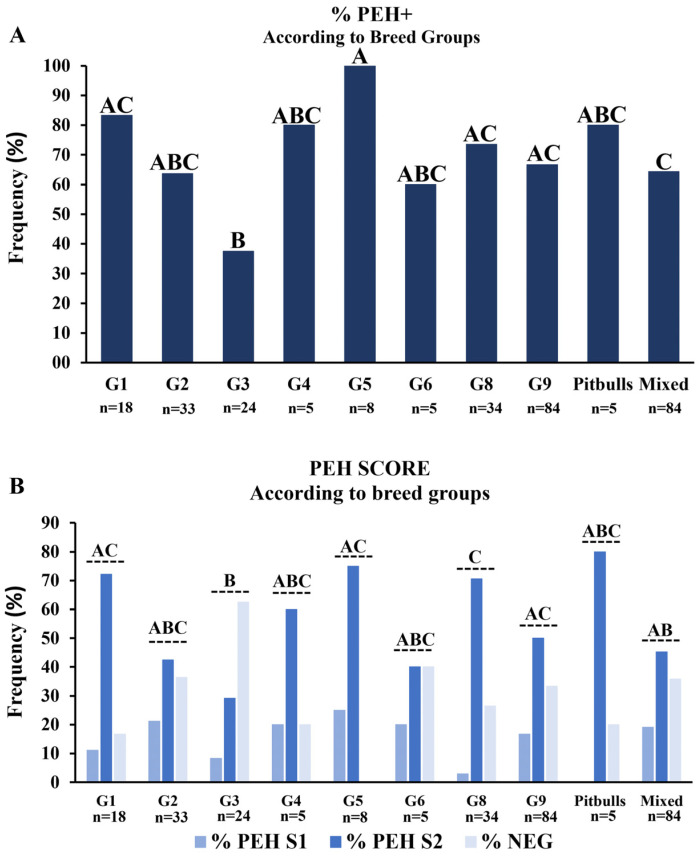
Frequencies and scores of pseudoplacentational endometrial hyperplasia (PEH) according to breed groups. (**A**) Frequencies of PEH+ cases according to breed groups, Pitbull, and mixed-breed dogs. (**B**) Score analyses of PEH according to breed groups: Pitbull and mixed-breed dogs. Medians of % PEH+ were compared by non-parametrical Fisher’s exact test and score distribution by Kruskal–Wallis and Dunn tests. Uppercase letters indicate statistical differences between age intervals (*p* < 0.05). G1: Herding and cattle dogs (Border Collie, German Shepherd, Swiss Shepherd, and White Swiss Shepherd); G2: Pinscher, Schnauzer, Molossoids, and Swiss Cattle Dogs (Bernese, Boxer, Bulldog, Fila Brasileiro, Pinscher, Rottweiler, Schnauzer, and Shar-Pei); G3: Terriers (Brazilian Terrier and Yorkshire); G4: Dachshund (Dachshund); G5: Spitz-type dogs and primitive-type dogs (Akita, Chow-Chow, Siberian Husky, Samoyed, and German Spitz); G6: Hounds and blood track dogs (Basset, Basset Hound, Beagle, and Dalmatian); G8: Retrievers, Hunt collecting/hunting dogs, and water dogs (Labrador Retriever, Golden Retriever, and Cocker); G9: Companion dogs (French bulldog, Chihuahua, Lhasa Apso, Maltese, Pekingese, Poodle, Pug, and Shih-Tzu).

Dogs were grouped according to the breed groups indicated by the FCI classification, and the frequency and score of PEH were analyzed. Mixed breeds and Pitbulls were classified as separate groups since they are not included in the FCI classification. The frequency of PEH was significantly lower in group 3, which includes Brazilian Terriers and Yorkshire than in groups 1 (herding and cattle dogs), 5 (Spitz and primitive type dogs), 8 (Retrievers), 9 (Companion dogs), and mixed-breed dogs (Figure 5A). Similar results were observed in the PEH score analyses, except that no significant difference was observed between group 3 and mixed-breed dogs (Figure 5B).

#### 3.2.2. Frequencies of CEH According to Age, Size, Breed, and Breed Group

**Figure 6:**
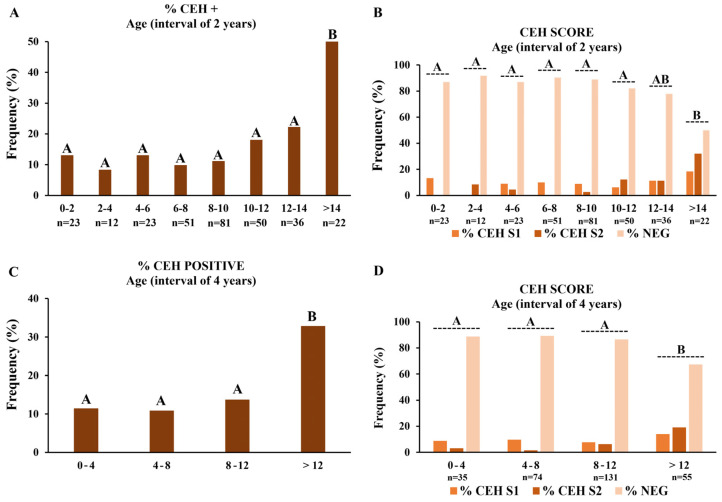
Frequencies and scores of cystic endometrial hyperplasia (CEH) according to age intervals. (**A**) Frequencies of CEH+ cases according to age intervals of two years. (**B**) Score analyses according to age intervals of two years. (**C**) Frequencies of CEH+ cases according to age intervals of four years. (**D**) Score analyses according to age intervals of four years. Medians of % CEH+ were compared by non-parametrical Fisher’s exact test, and score distributions in each age interval were compared by Kruskal–Wallis and Dunn tests. Uppercase letters indicate statistical differences between age intervals (*p* < 0.05).

Similar to the PEH, analyses were performed considering the occurrence of CEH, investigating predispositions of CEH according to age, size, breed, and breed groups. Grouping animals at 2-year intervals, both frequencies and scores of CEH positive (CEH+) cases were significantly higher in dogs older than 14 years (Figure 6A,B). Considering 4-year intervals, both the frequency and score of CEH were significantly higher in dogs older than 12 years (Figure 6C,D). Together, these results demonstrated that CEH is more frequent in dogs older than 12 years. There were no differences in the frequencies or score analyses of CEH considering different sizes, breeds, or breed groups (Supplementary Figure S2).

### 3.3. Frequencies of PEH and CEH in Dogs with Uterine Inflammation

**Figure 7:**
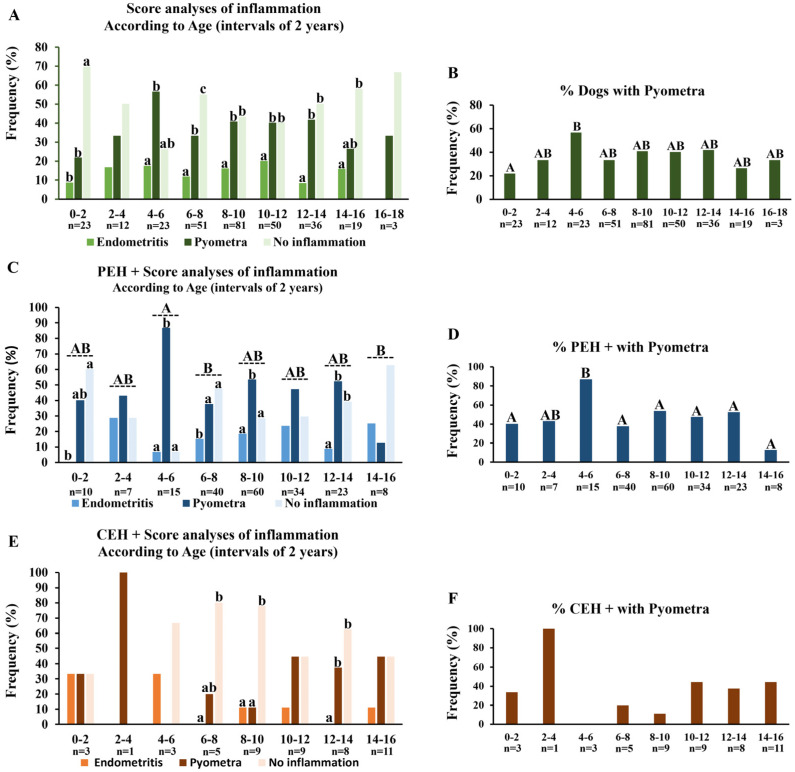
Analyses of uterine inflammation in all dogs included in this study: dogs with pseudoplacentational endometrial hyperplasia (PEH) and dogs with cystic endometrial hyperplasia (CEH) according to age intervals of two years. (**A**) Score classifications of the uteri of all dogs included in this study. Uterine inflammation analyses considered score classification, frequencies of pyometra, endometritis, and no inflammation in each age interval, and frequencies of pyometra between age intervals. (**B**) Frequency of pyometra in the uteri of all dogs included in this study. (**C**) Classification of uterine inflammation in dogs with PEH. (**D**) Frequency of pyometra in the uteri of dogs with PEH. (**E**) Classification of uterine inflammation in dogs with CEH. (**F**) Frequency of pyometra in the uteri of dogs with CEH. (**A**–**F**) Medians of frequencies were compared by non-parametrical Fisher’s exact test, and distributions of classifications were compared by Kruskal–Wallis and Dunn tests; uppercase letters indicate statistical differences (*p* < 0.05). Frequencies of no inflammation, endometritis, and pyometra were compared by Fisher’s exact test, and lowercase letters indicate statistical differences (*p* < 0.05).

Considering all female dogs, regardless of hyperplastic lesions, uterine inflammation was diagnosed in 52.3% (157/300), with 14.3% (43/300) of dogs with endometritis and 38.0% (114/300) with pyometra. Evaluating the distribution of uterine inflammation according to age interval of 2 years, frequencies of endometritis and pyometra at various age intervals are illustrated in Figure 7A. Importantly, the frequency of pyometra was significantly higher in dogs with 4–6 years of age compared to those 0–2 years old (Figure 7B). Frequencies of endometritis, uterus without inflammation, and pyometra in each age interval demonstrated that endometritis had lower frequencies than pyometra and uterus without inflammation at intervals between 6 and 14 years old, with differences between pyometra and uterus without inflammation only from 6 to 8 years old (Figure 7A).

Considering female dogs with PEH, inflammation was diagnosed in 64.8% (129/199) of the cases, with 16.1% (32/199) characterized as endometritis and 48.7% (97/199) characterized as pyometra. The frequency of pyometra was significantly higher in uteri with PEH than both frequency of endometritis (*p* < 0.0001) and uterus without inflammation (*p* = 0.0176). Conversely, endometritis was less frequent than uterus without inflammation in PEH-positive cases (*p* < 0.0001). The classification of inflammation was significantly different at from 4 to 6 than at from 6 to 8 and from 14 to 16 years old, probably due to a higher frequency of pyometra than endometritis and uterus without inflammation at from 4 to 6 years old (Figure 7C). Analyzing frequencies in each age interval, endometritis was less frequent than pyometra and uterus without inflammation at from 0 to 2, from 6 to 8, and from 12 to 14 years old (Figure 7C). Considering frequencies of pyometra between age intervals, it was more frequent in dogs aged from 4 to 6 years old than in all other age intervals except for in dogs aged from 2 to 4 years old (Figure 7D).

Considering dogs with CEH, uteri without inflammation were more frequent than uteri with endometritis (*p* < 0.0001) or pyometra (*p* = 0.032). Endometritis and pyometra were present in 10.2% (5/49) and 32.6% (16/49) of the cases, respectively, totaling 42.8% (21/49) of CEH cases with uterine inflammation. Classification (Figure 7F) and frequencies of pyometra (Figure 7E) were not significantly different among age intervals. Analyzing scores in each age interval, at from 8 to 10 and from 12 to 14 years, uterus without inflammation was more frequent than uterus with endometritis or pyometra and uterus with endometritis, respectively (Figure 7E).

## 4. Discussion

This study identified predisposing factors for PEH, such as age, size, and breed group. PEH was more frequent in dogs between 4 and 12 years of age. Since the pathogenesis of PEH is not well known, it is not possible to speculate on mechanisms leading to age predisposition. However, hormonal effects or reproductive characteristics, e.g., number of ovulations or number of pregnancies, must be considered once other reproductive conditions are influenced by these factors [1,7]. This study also demonstrated that frequencies of CEH were higher in dogs that were more than 12 years old, the same period of age in which PEH frequencies were reduced, corroborating the idea that these conditions are different and suggesting that they do not occur in the same period of dog life [3,5]. Yorkshires and the breeds in group 3, which include Brazilian Terriers and Yorkshires, were less often affected by PEH than Shih-Tzu and other breed groups. This observation suggests that breeds from group 3 can carry genetic or reproductive features that can make them less susceptible to the development of PEH. Although epidemiologic data on PEH were lacking prior to this study, there are many studies assessing dog breed predisposition to pyometra; however, results are highly impacted by study design and geographic location [12]. Importantly, this study may also have some bias due to inclusion criteria, i.e., part of the caseload of the veterinary hospital, which does not necessarily reflect the general canine population, among other factors, such as diet and incomplete records, may also impact the interpretation of the results. These are limiting factors for clinical studies in general [13]. However, the large number of animals included in this study minimizes the potential limitations of the experimental design. Furthermore, the frequency of breeds in this study reflects the local population, so results in this study may not quite reflect other geographic areas that have a different breed composition compared to the canine population [14]. Underrepresented breeds may prevent accurate analysis [15], which may also be influenced by breed-specific predisposing factors [16]

Our results support the notion that PEH is still underreported, probably due to a lack of knowledge and underdiagnoses of this condition. The results of this study demonstrated that PEH was more frequently diagnosed than CEH, which has also been demonstrated by our group in a previous study [5]. Uteri with PEH had a higher incidence of pyometra than endometritis or absence of inflammation, and pyometra was observed in dogs from all age intervals. Conversely, in uteri with CEH, the absence of inflammation was more frequent than both endometritis and pyometra. Together, these observations support the hypothesis that PEH may be a predisposing factor for pyometra, which is in good agreement with our previous study [5]. Furthermore, this study indicated that CEH is not significantly associated with pyometra, reinforcing the idea that the terminology “CEH pyometritic complex” and the four types of classification determined by Dow (1959) are no longer suitable [3,5,10]. Importantly, from a clinical point of view, the diagnosis of PEH may have a lower frequency when compared to histopathology, as the diagnostic standard, since only more severe cases are recognized clinically [17,18]

The frequency of pyometra in this study was higher at 4–6 years of age when compared to 0–2 years-old dogs, which is slightly different from multiple previous studies that observed a higher frequency of pyometra in dogs with from 6.50 to 9.36 years of age [6,11,19,20,21,22]. This is likely due to variation in inclusion criteria for those various studies since this study was based on a retrospective analysis of randomly selected samples and not focused on dogs developing clinical changes or other inclusion/exclusion criteria.

This study provides relevant epidemiologic data on PEH and CEH. However, these findings do not establish a cause-and-effect relationship between the parameters evaluated in this study and the occurrence of PEH or CEH, which should be addressed by future studies. Although there are a few studies addressing mechanisms leading to endometrial hyperplastic changes [14,23,24,25,26,27], the pathogeneses or PEH and CEH are still poorly known.

## 5. Conclusions

In conclusion, results from this study demonstrated predisposing factors for PEH, such as breed, breed group, size, and age, which need to be investigated and considered in PEH pathogenesis. Furthermore, PEH is more frequent than CEH and significantly associated with pyometra, although a cause-and-effect relationship between PEH and pyometra still needs to be addressed experimentally since analyses of frequencies or correlation cannot establish a causal effect.

## Figures and Tables

**Table 1:** Classification of breed groups according to Federation Cynologique Internationale (FCI—https://www.fci.be/en/ accessed on 22 December 2024) classifications and breeds from this study classified in each group.

Classification	Name of the Group	Breeds from This Study
Group 1	Herding and cattle dogs (except Swiss Cattle Dogs)	Border Collie, German Shepherd, Swiss Shepherd and White Swiss Shepherd
Group 2	Pinscher, Schnauzer, Molossoids and Swiss Cattle Dogs	Bernese, Boxer, Bulldog, Fila Brasileiro, Pinscher, Rottweiler, Schnauzer and Shar-Pei
Group 3	Terriers	Brazilian Terrier and Yorkshire
Group 4	Dachshund	Dachshund
Group 5	Spitz-type dogs and primitive-type dogs	Akita, Chow-Chow, Siberian Husky, Samoyed and German Spitz
Group 6	Hounds and blood track dogs	Basset, Basset Hound, Beagle and Dalmatian
Group 7	Continental Pointers and Pointers of the British Isles	No breed from this group in this study
Group 8	Retrievers, Hunt collecting/hunting dogs and water dogs	Labrador retriever, Golden Retriever and Cocker
Group 9	Companion dogs	French bulldog, Chihuahua, Lhasa Apso, Maltese, Pekingese, Poodle, Pug and Shih-tzu
Group 10	Greyhounds	No breed from this group in this study

**Table 2:** Histological characterization and scoring criteria of pseudoplacentational endometrial hyperplasia (PEH), cystic endometrial hyperplasia (CEH), and uterine inflammation (UI) adapted from Santana et al. (2020).

Lesion	Histological Characterization of Lesions	Score (S) of Intensity
PEH	Decidual reaction of luminal epithelium and superficial glandular epithelium with epithelial hyperplasia and hypertrophy, forming papillary projections to the lumen. Variable degrees of glandular cystic dilation.	S1: multifocal decidual reaction S2: diffuse decidual reaction
CEH	Endometrial superficial and deep glands with cystic dilatation homogeneously distributed in the endometrium. Superficial endometrium and glands with cuboidal to flattened and non-vacuolated epithelium.	S1: mild to moderate S2: severe
UI	Uterine inflammation was classified as endometritis or pyometra. Endometritis: interstitial infiltration of inflammatory cells, predominantly lymphocytes, plasma cells and histocytes. Rare neutrophils and eosinophils. Pyometra: interstitial infiltration of inflammatory cells, predominantly lymphocytes, plasma cells and histocytes. Marked intraluminal neutrophilic accumulation within endometrial lumen and/or glands, with variable degrees of neutrophilic exocytosis.	S1: mild interstitial inflammatory infiltrate (endometritis) S2: moderate interstitial inflammatory infiltrate (endometritis) S3: severe interstitial and luminal inflammatory infiltrate (pyometra)

## Data Availability

Data that support the findings described in this paper are contained within this manuscript.

## Supplementary Materials

The following supporting information can be downloaded at https://www.mdpi.com/article/10.3390/vetsci12010001/s1: Figure S1: Characterization of female dogs included in this study; Figure S2: Frequencies of cystic endometrial hyperplasia (CEH) in dogs and score analyses, with CEH score 1 (S1), CEH score 2 (S2) and negative CEH cases (NEG), according to breed and size.
