# Supplementary material for: Predisposing Factors for Pseudoplacentational Endometrial Hyperplasia or Cystic Endometrial Hyperplasia in Dogs and Their Association with Pyometra
Source: Vet Sci. 2024 Dec 26;12(1):1. doi: 10.3390/vetsci12010001 (PMC11768680; doi:10.3390/vetsci12010001)
Supplement: Supplementary file 1 [file vetsci-12-00001-s001.zip › Supplementary Figure S1.pdf]

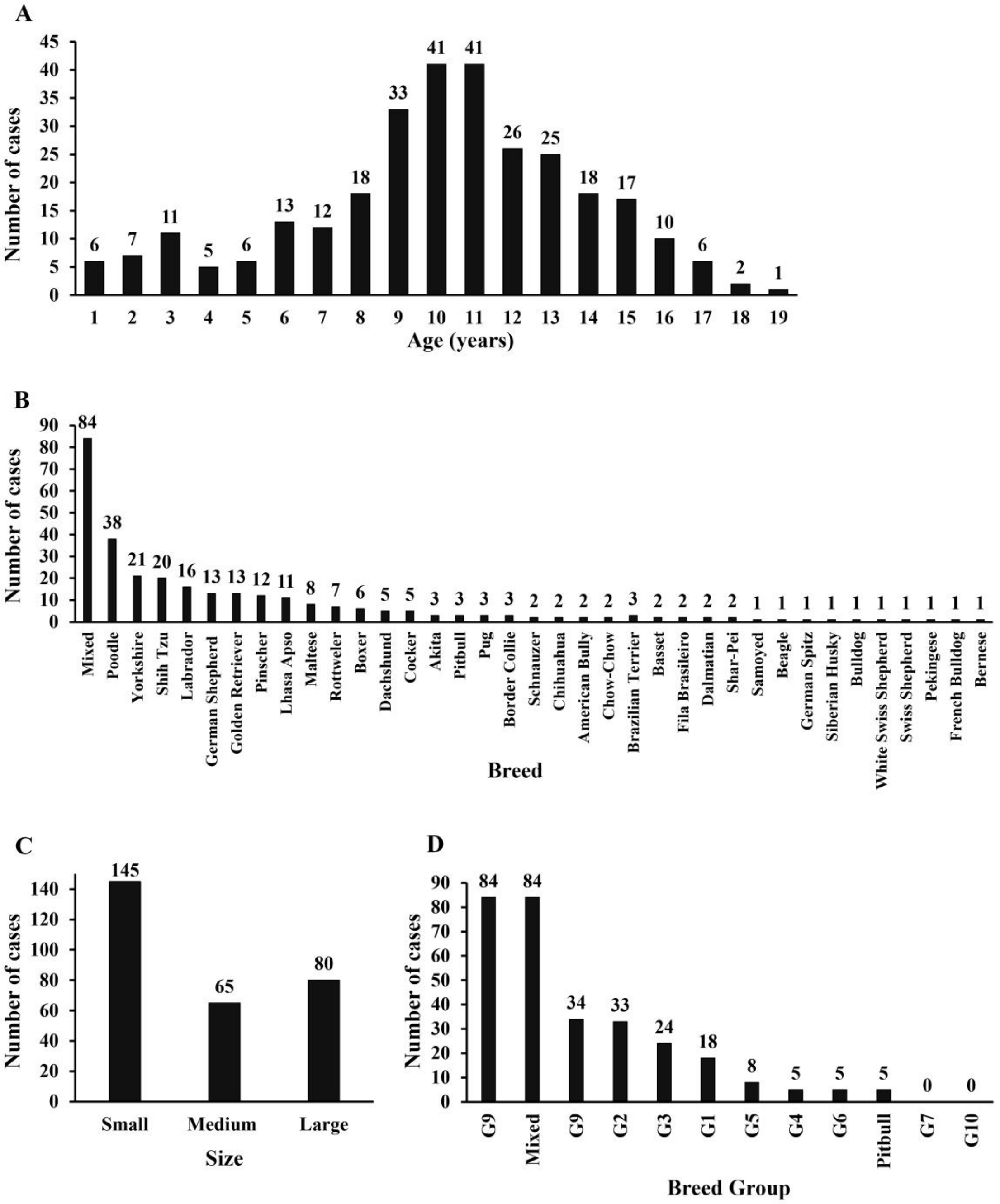

Supplementary Figure S1. Characterization of female dogs included in the study. (A) Number of female dogs included in the study according to age. (B) Number of female dogs included in the study according to breed. (C) Number of female dogs included in the study according to size. Size was classified including three categories: small, medium and large. Dogs were classified in each category of size considering the breed size or weigh, for mixed breed animals and breeds with variable size phenotype. (D). Number of female dogs included in the study according to breed groups classified considering the *Federation Cynologique Internationale* (FCI) classification. G1:Herding and cattle dogs (Border Collie, German Shepherd, Swiss Shepherd and White Swiss Shepherd), G2: Pinscher, Schnauzer, Molossoids and Swiss Cattle Dogs (Bernese, Boxer, Bulldog, Fila Brasileiro, Pinscher, Rottweiler, Schnauzer and Shar-Pei), G3: Terriers (Brazilian Terrier and Yorkshire), G4: Dachshund (Dachshund), G5: Spitz-type dogs and primitive-type dogs (Akita, Chow-Chow, Siberian Husky, Samoyed and German Spitz), G6: Hounds and blood track dogs (Basset, Basset Hound, Beagle and Dalmatian), Group 7: Continental Pointers and Pointers of the British Isles (no breed from this group in this study), G8: Retrievers, Hunt collecting/hunting dogs and water dogs (Labrador retriever, Golden retriever and cocker), G9:

Companion dogs (French bulldog, Chihuahua, Lhasa Apso, Maltese, Pekingese, Poodle, Pug and Shih-tzu) and G10: Greyhounds (no breed from this group in this study).
