# Supplementary material for: Predisposing Factors for Pseudoplacentational Endometrial Hyperplasia or Cystic Endometrial Hyperplasia in Dogs and Their Association with Pyometra
Source: Vet Sci. 2024 Dec 26;12(1):1. doi: 10.3390/vetsci12010001 (PMC11768680; doi:10.3390/vetsci12010001)
Supplement: Supplementary file 1 [file vetsci-12-00001-s001.zip › Supplementary Figure S2.pdf]

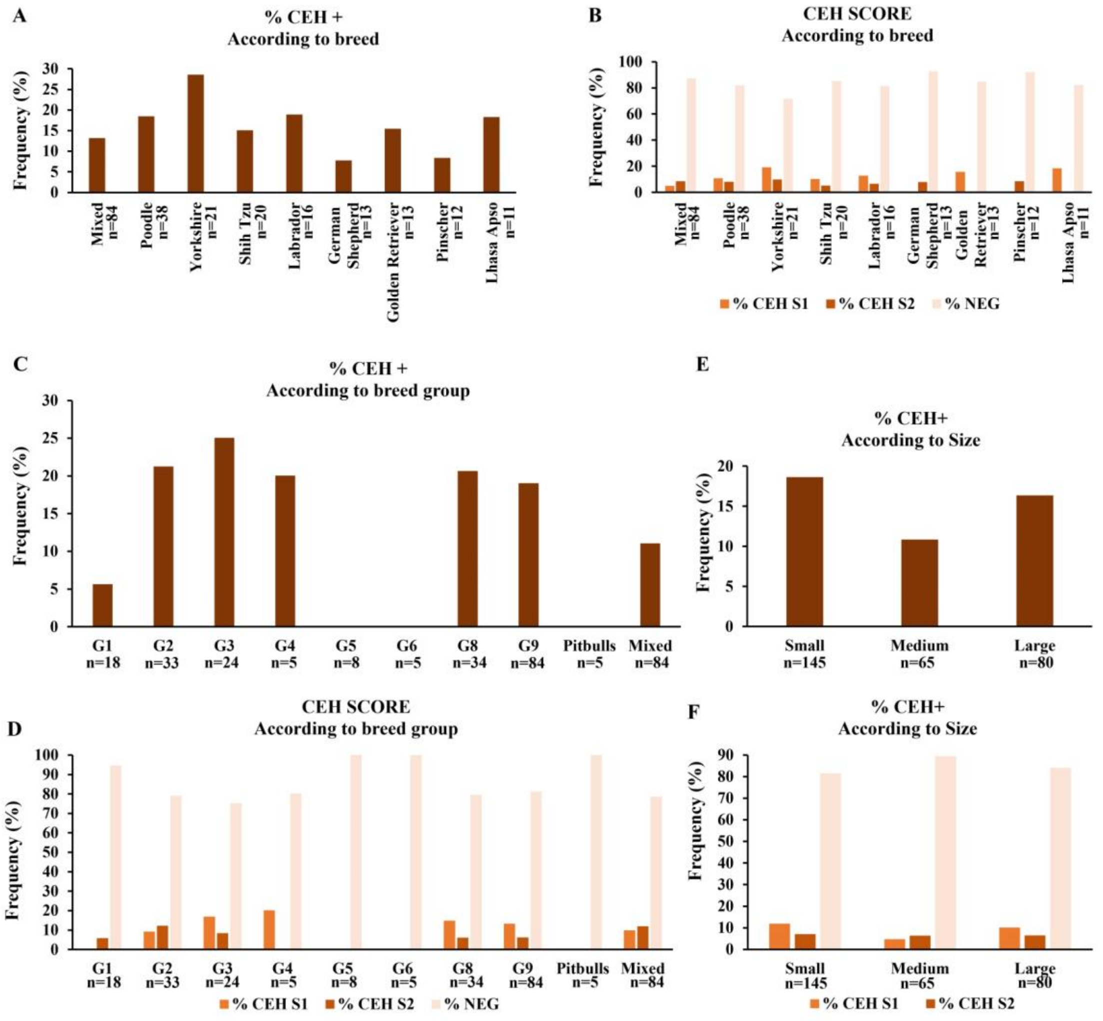

Supplementary Figure S2. Frequencies of cystic endometrial hyperplasia (CEH) in dogs and score analyses, with CEH score 1 (S1), CEH score 2 (S2) and negative CEH cases (NEG), according to breed and size. (A) Frequencies of CEH+ cases according to breeds. (B) Score analyses of CEH according to breeds. (C) Frequencies of CEH+ cases according to breed groups, Pitbull and mixed breed dogs. (D) Score analyses of PEH according to breed groups, Pitbull and mixed breed dogs. (E) Frequencies of CEH+ cases according to size including small, medium and large dogs. (F) Score analyses of CEH according to size. Medians of % CEH+ were compared by non-parametrical Fisher's exact test and score distribution were compared by Kruskal-Wallis and Dunn tests. No significant differences were observed ($p > 0.05$). G1:Herding and cattle dogs (Border Collie, German Shepherd, Swiss Shepherd and White Swiss Shepherd), G2: Pinscher, Schnauzer, Molossoids and Swiss Cattle Dogs (Bernese, Boxer, Bulldog, Fila Brasileiro, Pinscher, Rottweiler, Schnauzer and Shar-Pei), G3: Terriers (Brazilian Terrier and Yorkshire), G4: Dachshund (Dachshund), G5: Spitz-type dogs and primitive-type dogs (Akita, Chow-Chow, Siberian Husky, Samoyed and German Spitz), G6: Hounds and blood track dogs (Basset, Basset Hound, Beagle and Dalmatian), G8: Retrievers, Hunt collecting/hunting dogs and water dogs (Labrador retriever, Golden retriever and cocker), G9: Companion dogs (French bulldog, Chihuahua, Lhasa Apso, Maltese, Pekingese, Poodle, Pug and Shih-tzu).
